# Impact of oral health conditions on the quality of life of *quilombola* and non-*quilombola* rural adolescents in the countryside of Bahia, Brazil: a cross-sectional study

**DOI:** 10.1186/s12955-020-01567-x

**Published:** 2020-09-29

**Authors:** Etna Kaliane Pereira da Silva, Danielle Souto de Medeiros

**Affiliations:** Instituto Multidisciplinar em Saude, Campus Anisio Teixeira, Universidade Federal da Bahia. Rua Hormindo Barros, 58, quadra 17, lote 58, Candeias, Vitória da Conquista, BA 45029-094 Brazil

**Keywords:** Oral health, Quality of life, Adolescents, Rural health, African continental ancestry group

## Abstract

**Background:**

This study aimed to estimate the prevalence of negative impacts of oral health conditions on the quality of life of *quilombola* and non-*quilombola* rural adolescents and identify associated factors.

**Methods:**

This cross-sectional study was carried out in a rural area in the countryside of Bahia, Brazil, in 2015. Participants were asked to complete the Oral Impacts on Daily Performance Questionnaire. Prevalence and prevalence ratios (PR) were estimated together with their respective 95% confidence intervals. Multiple analysis was conducted using Poisson regression with robust error variance and hierarchical entry of variables.

**Results:**

Of the 390 rural adolescents who took part in the study, 42.8% were *quilombolas*, and 45.6% of all participants reported a negative impact of their oral health conditions on their quality of life. The most prevalent impact was difficulty eating (32.6%). After adjusted analysis, the following factors were found to be associated with the negative impact of oral health conditions on quality of life: age (PR = 1.04), feeling lonely (PR = 1.42), worst evaluation of oral health (PR = 1.52), need of dental care (PR = 1.33), and occurrence of toothache in the last 6 months (PR = 1.83). *Quilombolas* and non-*quilombolas* presented with a different prevalence of discomfort when brushing their teeth and had different factors associated with the negative impact of oral health conditions on their quality of life. Both *quilombola* and non-*quilombola* rural adolescents showed a high prevalence of negative impact of oral health conditions on their quality of life.

**Conclusions:**

These results support the need for improved oral healthcare for specific populations like the *quilombolas*. Furthermore, the results illustrate the importance of incorporating oral healthcare strategies that take into consideration the sociocultural context of adolescents.

## Background

Oral health is integral to general health and is a determinant factor for quality of life [1]. For adolescents, associations between oral health, general health, and quality of life are strongest, as this group is the most sensitive to the negative impacts of oral health conditions on a range of aspects, including the perception of physical appearance and pain [2].

Oral health conditions can interfere with the psychological development and social interactions of adolescents [3]. Also, daily activities can be affected. For example, adolescents may experience discomfort when brushing their teeth and difficulty eating and/or speaking [2]. Socio-dental indicators are often used to measure the negative impact of oral health conditions on quality of life because they reflect individuals’ self-perceptions and because clinical criteria alone do not permit a holistic evaluation of the impact of oral health conditions on daily life [4].

A study of Brazilian urban adolescents between 15 and 19 years of age found that 39.4% reported at least one negative impact of oral health conditions on daily life [2]. A higher prevalence of negative impact was found in international studies, varying from 54.6 to 66.8% [5–7]. This impact in the quality of life of adolescents has been associated not only with oral diseases and complications, like untreated cavities, tooth loss, toothache, periodontal disease, and malocclusion, but also with individual characteristics, including gender, race, and economic conditions [2, 3, 8].

Hardships, such as difficulty accessing education and health services and unfavourable socioeconomic conditions, can influence an individual’s oral health status and healthcare utilisation, which can in turn negatively impact their quality of life. Adolescents who live in rural areas are exposed to different cultural influences and are subject to social vulnerabilities and health inequities. Among them, there are groups, like *quilombola* adolescents, with greater vulnerability [9].

*Quilombolas* are traditional Brazilian people with a presumption of black ancestry who keep alive their cultural and religious traditions, kinship, and identity marked by resistance to oppression, the denial of their rights, and racial segregation. *Quilombola* adolescents have a lower prevalence of dental consultations when compared to other rural adolescents. In addition, they reside in communities with greater social vulnerabilities, such as a precarious sanitised water supply, irregular garbage collection, low education level, and low family income [10, 11].

Studies on the negative impact of oral health conditions on adolescents’ quality of life do not include adolescents who live in rural areas or traditional communities, like *quilombola* communities. Considering that the living environment influences individuals’ oral health [12], this study aimed to estimate the prevalence of negative impact of oral health conditions on quality of life and identify associated factors in *quilombola* and non-*quilombola* adolescents in a rural area in the countryside of Bahia, Brazil.

## Methods

This cross-sectional and population-based study was part of the research project *ADOLESCER: Saude do Adolescente da Zona Rural e seus Condicionantes*, which was carried out in 2015. Subjects were adolescents between 10 and 19 years of age who lived in 21 *quilombola* rural communities, all of them recognised by the *Fundacao Cultural Palmares,* and non-*quilombola* rural communities in Vitoria da Conquista, Bahia, Brazil.

The research was approved by the ethics committee for research on human beings in the Multidisciplinary Institute of Health of Bahia Federal University (*Comite de Etica em Pesquisa com seres humanos do Instituto Multidisciplinar em Saude da Universidade Federal da Bahia*) under report number 639.966. All of the participants were previously informed on the research goals, procedures, and data confidentiality through the Free and Informed Consent Form (*Termo de Consentimento/Assentimento Livre e Esclarecido*) and expressed their agreement to participate in this study through signature or automated fingerprint identification.

To carry out the population estimate, we collected data from Brazilian Primary Health Care forms, used by the community health workers during the household visits. At the time of the study, 97.4% of the rural area of Vitoria da Conquista was covered by the Program of Community Health Workers.

To ensure viability and representativeness of the research, the sampling strategy took into account the territorial extension of rural communities and populations of adolescents. The sampling principles used were as follows: (1) the number of homes was selected proportionally to the number of adolescents per community, and (2) only one adolescent was interviewed per home. Moreover, in order to obtain valid estimates for *quilombola* and non-*quilombola* populations, the sample size was calculated separately for each stratum.

The sample size calculation was carried out using the following criteria: a prevalence of 50%, given the heterogeneity of the events measured in the main project; an accuracy of 5%; a confidence level of 95%; a design effect equal to 1.0; and an addition of 15% for possible losses. However, as only one adolescent per home was interviewed, and because the number of homes was smaller for the *quilombola* communities, 7.1% of losses were added to the *quilombola* stratum. The presence of severe mental disorders among adolescents was used as an exclusion criterion.

Sampling for non-*quilombola* adolescents was carried out in two stages: (1) random selection of homes with adolescents, according to the proportional distribution of adolescents per community, and (2) random selection of adolescents in each home. For the *quilombola* sample, only random selection of adolescents in each home was used. All adolescents had the same probability of inclusion in the study.

The instrument used for the interview was a questionnaire with objective questions that have been described in previous surveys conducted in Brazil: National Oral Health Survey (*Pesquisa Nacional de Saude Bucal*, *SBBrasil*) [13], National School Health Survey (*Pesquisa Nacional de Saude do Escolar*, *PeNSE*) [14], and Health National Research National Health Survey (*Pesquisa Nacional de Saude, PNS*) [15]. The questionnaire was divided in two sections: (1) the first section was answered by the adolescents (≥ 18 years) or their parents/guardians, and had questions about the general characteristics of their household and income and education of the head of the family; (2) the second section was answered by the investigated adolescent; it had questions about their characteristics and social support, job, lifestyle, perception of health status and body self-image, use of illicit drugs, sexual and reproductive health, oral health and hygiene, and use of health services.

The instrument underwent occasional modifications for adaptation in rural contexts; however, the original structure of the validated questionnaires was maintained to guarantee the reliability and comparability of the information. The final version was submitted to a pre-test and a pilot study to adapt to the following: (1) language (adaptations in terms of vocabulary); (2) sequence and coherence between the questions; (3) instructions on the questions to be skipped; and (4) the time required to conduct the interview.

Before data collection, the households and social facilities of the communities were mapped and the residents were sensitized to the research. The sample collection took place between January and May 2015. To guarantee the quality of the collected data, a second interview was conducted with 5% of the sample within 7 days of the first interview.

The outcome variable of this study was the negative impact of oral health conditions on quality of life, and it was measured using the Brazilian version used in the National Oral Health Survey [13] of Oral Impact on Daily Performance (OIDP) index [16]. This version was translated, adapted, and validated for use in Brazilian Portuguese. It has a Cronbach's α of 0.78 [95% confidence interval (CI) 0.77–0.79] and has been used in Brazilian population-based studies [2, 17–19].

The OIDP assesses the impact of oral health conditions on eight daily activities, which cover physical (difficulty to eat, discomfort when brushing tooth, difficulty to speak), psychological (sleeping troubles, embarrassment when smiling, nervousness, and irritation), and social (problems with studying, interference in leisure) dimensions [16]. Each item was preceded by the question, ‘Some people have problems that may have been caused by their teeth. From the situations given below, which ones apply to you in the last six months?’ The answer provided were no and yes [16]. The OIDP was analysed as a dichotomous variable: absence of impact (total OIDP = 0) or presence of impact (total OIDP ≥ 1). A positive answer to at least one of the eight items of the index was considered “yes”. We analysed the OIDP as a dichotomous variable because the distribution of the OIDP scores demonstrated two distinct groups. In addition, the conceptual approach supports this cut-off point, which has been used in previous studies with adolescent populations [5, 18, 20].

**Fig. 1:**
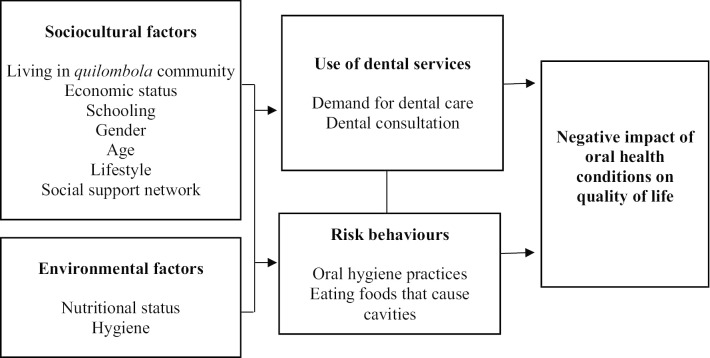
Conceptual model for multiple analysis, adapted according to Petersen [21]

The independent variables of this study were selected based on the conceptual model proposed by Petersen [21], in which the first section is formed by sociocultural and environmental factors and the second section by variables related to the utilisation of dental services and oral health risk behaviours (Fig. 1).

The chosen sociocultural characteristics were as follows: living in a *quilombola* community (yes; no), economic level (B and C—higher levels; D and E—lower levels) [22]; gender (female; male); age (years); schooling (years of study); tobacco use (no; yes); illicit drug use (no; yes); practice of physical exercise (< 300 min/week; ≥ 300 min/week); close friends (up to 2 friends; more than 3 friends); family composition (adolescents living with their parents; adolescents not living with their parents); feelings of loneliness in the last 12 months (never/rarely; sometimes; often/always); and parents who were understanding of their personal issues in the last 30 days (often or always; sometimes; never or rarely).

The chosen environmental factors were as follows: the nutritional status obtained through standard measures for weight and height and body mass index (BMI) classified according to gender and age (thinness; eutrophy; overweight/obesity) [23]; and handwashing before meals (often or always; sometimes; never/rarely).

To characterise the utilisation of dental services, the following variables were used: self-assessment of oral health (very good/good; regular; bad/very bad); perception of a need for dental treatment (no; yes); toothache in the last 6 months (no; yes); and dental consultation in the last year (yes; no). In addition, oral health risk behaviours considered were: tooth brushing less than 3 times a day; no use of dental floss; regular consumption (≥ 5 days per week) of unhealthy snacks and soft drinks.

The prevalence of negative impacts on each daily performance and the occurrence of at least one negative impact of oral health conditions on quality of life were evaluated for non*-quilombolas, quilombolas,* and the total sample. Prevalence differences between *quilombolas* and non-*quilombolas* were evaluated using the Pearson chi-squared test. For all tests, a significance level of 5% (*P* < 0.05) was considered statistically significant.

Prevalence ratios and their respective 95% confidence intervals, as estimated by Poisson regression with robust variance, were used to identify variables associated with the occurrence of negative impact of oral health conditions on quality of life in the total sample and the non-*quilombola* and *quilombola* samples. This model was chosen because it directly estimates the prevalence ratios, which is more appropriate due to the high prevalence of the analysed outcome [24]. All variables with a significance level of 20% in the bivariable analysis were included in the multiple analysis.

The multiple analysis was conducted using a hierarchical entry of the variables, initially including the set of sociocultural and environmental variables and later the set of variables related to the use of dental services and oral health risk behaviours. The variables of the first set were used as adjustment factors for the second set of variables. To compare the models, the Akaike information criterion (AIC) and Bayesian information criterion (BIC) were used, and the adjustment of the predicted values to the observed values was evaluated by the chi-squared test. The Stata software version 15.0 (*Stata Corporation, College Station*, USA) was used for data analysis.

## Results

Of the 390 adolescents who were interviewed, 42.8% lived in *quilombola* communities. Differential losses were observed in relation to gender, with greater predominance in males between non-*quilombola* adolescents (*P* = 0.038). However, estimates with and without the calibration factor showed no significant differences. Thus, the analyses were conducted without considering the differential losses.

Most of the adolescents who participated in the survey had lower economic statuses and were female, physically inactive, had three or more close friends, lived with both parents, never or rarely felt alone, were mostly understood by their parents, were eutrophic, had a habit of washing their hands before meals, considered their oral health to be good or very good, consulted a dentist during the past year, and did not try tobacco or illicit drugs (Table 1).

**Table 1:** Descriptive characteristics of participants in the total sample, *quilombola* and non-*quilombola,* stratified according to negative impact of oral health condition on the quality of life. Bahia, 2015

Variables	Total sample			Non-*quilombola*			*Quilombola*		
	N (390)	Impact^a^		N (223)	Impact^a^		n (167)	Impact^a^	
		No (212)	Yes (178)		No (117)	Yes (106)		No (95)	Yes (72)
Sociocultural factors									
Economic status (%)									
B and C	38.7	35.8	42.1	49.8^¶^	46.1	53.7	23.9^¶^	23.2	25.0
D and E	61.3	64.2	57.9	50.2^¶^	53.9	46.2	76.0^¶^	76.8	75.0
Gender (%)									
Female	51.3	50.0	52.8	48.9	47.9	50.0	54.5	52.6	56.9
Male	48.7	50.0	47.2	51.1	52.1	50.0	45.5	47.4	43.1
Age, (median)^b^	14.8	14.3	15.3	14.6	14.5	14.7	15.0	14.2	16.0
Schooling (median)^c^	6.0	6.0	7.0	7.0	7.0	7.0	6.0	6.0	7.0
Trying tobacco (%)	5.1	3.3	7.3	5.4	3.4	7.5	4.8	3.2	6.9
Trying illicit drugs (%)	1.8	1.9	1.7	2.2	1.7	2.8	1.2	2.1	0.0
Practice of physical activities (%)									
Less than 300 min/week	54.1	57.5	50.0	54.7	58.1	50.9	53.3	56.8	48.6
More or equal 300 min/week	45.9	42.4	50.0	45.3	41.9	49.1	46.7	43.2	51.4
Close friends (%)									
Up to 2 friends	16.9	17.0	16.8	17.5	17.9	17.0	16.2	15.8	16.7
3 or more	83.1	83.0	83.2	82.5	82.1	83.0	83.8	84.2	83.3
Family composition (%)									
Live with parents	67.7	69.3	65.7	70.4	71.8	68.9	64.1	66.3	61.1
Do not live with parents	32.3	30.7	34.3	29.6	28.2	31.1	35.9	33.7	38.9
Feeling lonely (%)									
Never or rarely	63.3	70.3	55.7	62.8	70.1	54.7	64.1	70.5	64.1
Sometimes	29.0	23.1	36.0	30.9	23.1	39.6	26.3	23.1	26.3
Often/always	7.7	6.6	9.0	6.3	6.8	5.7	9.6	6.3	9.6
Parents understanding their issues (%)									
Often or always	42.3	42.1	42.6	42.5	41.2	43.8	42.2	43.2	40.8
Sometimes	32.2	32.5	31.8	32.4	36.0	28.6	31.9	28.4	36.6
Never or rarely	25.4	25.4	25.6	25.1	22.8	27.6	25.9	28.4	22.5
Environmental factors									
Nutritional status (%)									
Eutrophy	78.0	76.1	80.1	76.8	73.7	80.2	79.5	79.1	80.0
Thinness/malnutrition	3.4	2.9	4.0	4.1	3.5	4.7	2.5	2.2	2.9
Overweight/obesity	18.6	21.0	15.9	19.1	22.8	15.1	18.0	18.6	17.1
Handwashing before meals (%)									
Often or always	69.7	73.1	65.7	71.7	73.5	69.8	67.1	72.6	59.7
Sometimes/never/rarely	30.3	26.9	34.3	28.2	26.5	30.2	32.9	27.4	40.3
Use of dental services									
Oral health self-assessment (%)									
Very good/good	63.0	70.1	54.5	64.9	67.2	62.3	60.5	73.7	43.1
Regular	31.4	28.0	35.4	30.6	30.2	31.1	32.3	25.3	41.7
Bad/very bad	5.7	1.9	10.1	4.5	2.6	6.6	7.2	1.0	15.3
The need for dental treatment (%)	48.1	38.4	59.5	46.8	39.6	54.7	49.7	36.8	66.7
Toothache in the last six months (%)	19.6	8.1	33.3	18.5	8.6	29.2	21.1	7.4	39.4
Dental consultation in the last year (%)									
Yes	66.8	69.8	63.5	68.4	70.7	66.0	64.7	68.4	59.7
No	33.2	30.3	36.5	31.5	29.3	34.0	35.3	31.6	40.3
Oral health risk behaviour									
Toothbrushing (%)									
More than 3 times a day or equal	66.7	67.0	66.3	67.3	65.0	69.8	65.9	69.5	61.1
Less than 3 times a day	33.3	33.0	33.7	32.7	35.0	30.2	34.1	30.5	38.9
No use of dental floss (%)	46.7	44.8	48.9	46.2	44.4	48.1	47.3	45.3	50.0
Regular consume of snacks (%)	35.1	33.0	37.6	33.2	32.5	34.0	37.7	33.7	43.1
Regular consume of soft drinks (%)	6.7	7.1	6.2	7.2	5.9	8.5	6.0	8.4	2.8

^¶^*P* value < 0.05 calculated using Χ2 test (categorical variables) for the comparison between non-*quilombola* and *quilombola* samples

^a^Negative impact of oral health condition on the quality of life: No, absence of impact (total OIDP score = 0); Yes, presence of impact (total OIDP score ≥ 1)

^b^Age, Interquartile Range: Total Sample (total = 4.8; impact: no = 4.5; yes = 5.1); Non-*Quilombola* (total = 4.9; impact: no = 4.7; yes = 5.0); *Quilombola* (total = 4.6; impact: no = 4.2; yes = 4.2)

^c^Schooling, Interquartile Range: Total Sample (total = 4.0; impact: no = 4.0; yes = 4.0); Non-*Quilombola* (total = 4.0; impact: no = 4.0; yes = 4.0); *Quilombola* (total = 3.0; impact: no = 3.0; yes = 4.0)

However, 48.1% of the participants stated the need for dental treatment, and 19.6% reported dental pain in the last 6 months. Regarding oral health risk behaviours, 33.3% brushed their teeth fewer than three times a day; 46.7% did not floss; and 35.1% and 6.7% regularly consumed sweets and sodas, respectively. Significant differences between non-*quilombolas* and *quilombolas* were observed only in their economic status level (Table 1).

**Fig. 2:**
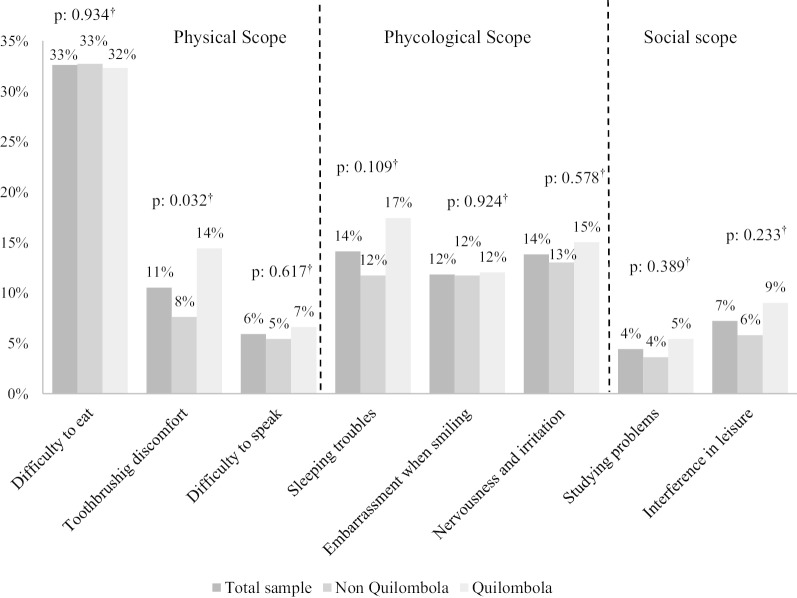
Prevalence of negative impacts in the total sample, non-*quilombola,* and *quilombola*. Bahia, 2015. ^†^*P* value calculated using the Pearson chi-square test for the comparison between non-*quilombola* and *quilombola* samples

The prevalence of negative impacts of oral health conditions among rural adolescents were difficulty eating (32.6%), sleeping troubles (14.1%), nervousness and irritation (13.8%), embarrassment when smiling (11.8%), and tooth brushing discomfort (10.5%). Differences among samples were observed only in tooth brushing discomfort, which was reported by 14.4% and 7.6% of *quilombola* and non-*quilombola* adolescents, respectively (Fig. 2). The overall prevalence of at least one negative impact of oral health conditions on quality of life was 45.6%, of which 47.5% was among non-*quilombola* adolescents and 43.1% was among *quilombola* adolescents. No significant difference in prevalence of at least one negative impact of oral health conditions on quality of life was found among *quilombolas* and non-*quilombolas*.

The prevalence of negative impacts of oral health conditions on quality of life was significantly higher among older adolescents, who reported feeling lonely sometimes over the past year, considered their oral health to be regular, bad, or very bad, and reported a need for dental treatment and the occurrence of toothache in the last 6 months (Table 2).

**Table 2:** Prevalence, prevalence ratio and 95% confidence interval of negative impact of oral health condition on the quality of life for the total sample, *quilombola* and non-*quilombola*. Bahia, 2015

Variable	Total sample			Non-*quilombola*			*Quilombola*		
	*P* (%)^†^	PR	95% CI	*P* (%)^†^	PR	95% CI	*P* (%)^†^	PR	95% CI
Sociocultural factors									
Economic status									
B and C	49.7	1.00	–	51.3	1.00	–	45.0	1.00	–
D and E	43.1	0.87	0.70–1.08	43.7	0.85	0.64–1.12	42.5	0.94	0.63–1.41
Gender									
Female	47.0	1.00	–	48.6	1.00	–	45.0	1.00	–
Male	44.2	0.94	0.76–1.17	46.5	0.96	0.72–1.26	40.8	0.90	0.63–1.29
Age	–	1.05	1.01–1.09	–	1.01	0.96–1.06	–	1.10	1.03–1.17
Schooling	–	1.02	0.98–1.06	–	0.99	0.94–1.04	–	1.08	1.01–1.15
Trying tobacco									
No	44.6	1.00	–	46.4	1.00	–	42.1	1.00	–
Yes	65.0	1.46	1.03–2.05	66.7	1.43	0.94–2.20	62.5	1.48	0.84–2.62
Trying illicit drugs									
No	45.7	1.00	–	47.2	1.00	–	43.6	1.00	–
Yes	42.9	0.94	0.39–2.22	60.0	1.27	0.61–2.64	0.0	–	–
Practice of physical activities									
Less than 300 min/week	42.3	1.00	–	44.3	1.00	–	39.3	1.00	–
More or equal 300 min/week	49.7	1.18	0.95–1.46	51.5	1.20	0.85–1.71	47.4	1.16	0.88–1.53
Close friends									
Up to 2 friends	45.4	1.00	–	46.1	1.00	–	44.4	1.00	–
3 or more	45.7	1.00	0.75–1.34	47.8	1.04	0.71–1.50	42.7	0.96	0.61–1.53
Family composition									
Live with parents	44.3	1.00	–	46.5	1.00	–	41.1	1.00	–
Do not live with parents	48.4	1.09	0.87–1.37	50.0	1.07	0.80–1.44	46.7	1.13	0.80–1.62
Feeling lonely									
Never or rarely	39.7	1.00	–	41.4		–	37.4	1.00	–
Sometimes	56.6	1.43	1.14–1.78	60.8	1.46	1.12–1.93	50.0	1.34	0.91–1.96
Often/always	53.3	1.34	0.93–1.94	42.9	1.03	0.55–1.96	62.5	1.67	1.06–2.63
Parents understanding their issues									
Often or always	46.0	1.00	–	49.5	1.00	–	41.4	1.00	–
Sometimes	45.2	0.98	0.76–1.27	42.2	0.85	0.61–1.20	49.1	1.18	0.80–1.75
Never or rarely	45.9	0.99	0.76–1.31	52.7	1.07	0.77–1.47	37.2	0.90	0.56–1.45
Environmental factors									
Nutritional status									
Eutrophy	47.5	1.00	–	50.3	1.00	–	43.7	1.00	–
Thinness/malnutrition	53.8	1.13	0.67–1.90	55.6	1.10	0.60–2.02	50.0	1.14	0.42–3.11
Overweight/obesity	39.4	0.83	0.61–1.13	38.1	0.76	0.50–1.15	41.4	0.94	0.59–1.52
Handwashing before meals									
Often or Always	43.0	1.00	–	46.2	1.00	–	38.4	1.00	–
Sometimes/never/rarely	51.7	1.20	0.96–1.50	50.8	1.09	0.82–1.47	52.7	1.37	0.97–1.94
Use of dental services									
Oral health self-assessment									
Very good/good	39.6	1.00	–	45.8	1.00	–	30.7	1.00	–
Regular	51.6	1.30	1.03–1.64	48.5	1.06	0.78–1.44	55.6	1.81	1.24–2.64
Bad/very bad	81.8	2.07	1.61–2.65	70.0	1.53	0.98–2.38	91.7	2.99	2.12–4.20
The need for dental treatment									
No	35.6	1.00	–	40.7	1.00	–	28.6	1.00	–
Yes	56.7	1.59	1.27–1.99	55.8	1.37	1.04–1.81	57.8	2.02	1.37–2.98
Toothache in the last 6 months									
No	37.8	1.00	–	41.4	1.00	–	32.8	1.00	–
Yes	77.6	2.05	1.70–2.47	75.6	1.82	1.43–2.33	80.0	2.44	1.81–3.28
Dental consultation in the last year									
Yes	43.5	1.00	–	46.0	1.00	–	39.8	1.00	–
No	50.4	1.16	0.93–1.44	51.4	1.12	0.84–1.49	49.1	1.23	0.87–1.75
Oral health risk behaviour									
Toothbrushing									
More than 3 times a day or equal	45.3	1.00	–	49.3	1.00	–	40.0	1.00	–
Less than 3 times a day	46.1	1.02	0.81–1.28	43.8	0.89	0.65–1.21	49.1	1.23	0.86–1.74
Use of dental floss									
Yes	43.7	1.00	–	45.8	1.00	–	40.9	1.00	–
No	47.8	1.09	0.88–1.36	49.5	1.08	0.83–1.42	45.6	1.11	0.78–1.58
Regular consume of snacks									
No	43.9	1.00	–	47.0	1.00	–	39.4	1.00	–
Yes	48.9	1.11	0.89–1.39	48.6	1.03	0.77–1.38	49.2	1.25	0.88–1.76
Regular consume of soft drinks									
No	45.9	1.00	–	46.7	1.00	–	44.6	1.00	–
Yes	42.3	0.92	0.58–1.46	56.2	1.20	0.76–1.89	20.0	0.44	0.13–1.57

*PR* prevalence ratio, *95% CI* 95% confidence interval

^†^*P*: prevalence of negative impact of oral health condition on the quality of life

Among non-*quilombola* adolescents, the most prevalent negative impact was among those who felt lonely sometimes, needed dental treatment, and had toothache in the last 6 months. Among *quilombolas*, a significant positive relationship was found with age, better schooling, worse oral health self-assessment, the need for dental treatment, and occurrence of toothache in the last 6 months (Table 2).

After multiple analysis, the following factors were found to be independently associated with the prevalence of negative impact of oral health conditions on quality of life in the total sample: age (PR = 1.04); feeling lonely sometimes (PR = 1.42); oral health self-assessment as bad or very bad (PR = 1.52); need for dental treatment (PR = 1.33); and the occurrence of toothache in the last 6 months (PR = 1.83) (Table 3).

**Table 3:** Factors associated with the negative impact of oral health condition on quality of life, according to the regression model for the total sample*,* non-*quilombola* and *quilombola*. Bahia, 2015

Variable	Total sample		Non*-quilombola*		*Quilombola*	
	PR	95% CI	PR	95% CI	PR	95% CI
Age	1.04	> 1.00–1.08	–	–	1.10	1.03–1.76
Feeling lonely						
Never or rarely	1.00	–	1.00	–	–	–
Sometimes	1.42	1.14–1.77	1.46	1.12–1.93	–	–
Often/always	1.30	0.89–1.88	1.03	0.55–1.96	–	–
Oral health self-assessment						
Very good/good	1.00	–	–	–	1.00	–
Regular	1.10	0.88–1.39	–	–	1.40	0.97–2.02
Bad/very bad	1.52	1.16–1.99	–	–	1.91	1.26–2.88
The need for dental treatment						
No	1.00	–	–	–	1.00	–
Yes	1.33	1.06–1.68	–	–	1.49	1.01–2.19
Toothache in the last 6 months						
No	1.00	–	1.00	–	1.00	–
Yes	1.83	1.51–2.22	1.75	1.37–2.24	1.83	1.32–2.52

*PR* prevalence ratio, *95% CI* 95% confidence interval

Among non-*quilombolas*, the prevalence of negative impact of oral health conditions on quality of life was independently associated with feeling lonely sometimes (PR = 1.46) and the occurrence of toothache in the last 6 months (PR = 1.75). Among *quilombolas*, the prevalence remained positively associated with age (PR = 1.10), oral health self-assessment as bad or very bad (PR = 1.91), need for dental treatment (PR = 1.49), and the occurrence of toothache in the last 6 months (PR = 1.83) (Table 3).

## Discussion

The rural adolescents in this study, including *quilombolas* and non-*quilombolas*, presented a high prevalence of negative impact of oral health conditions on quality of life. Sociocultural factors and related to the need for dental treatment presented the strongest association with the occurrence of negative impact.

However, a higher prevalence of negative impact of oral health conditions on quality of life has previously been reported among 12-year-old adolescents who participated in research in another region of Brazil (58.1%) [25], Italian pupils between 11 and 16 years of age (66.8%) [6], rural and urban adolescents in Uganda (62.0%) [5], and 12-year-old students in Sudan (54.6%) [7]. Such differences can be attributed to the local epidemiologic patterns of oral diseases.

Difficulty eating was the impact most frequently reported by rural adolescents, as found in many other studies [2, 7, 25, 26]. Importantly, around 20% of the adolescents interviewed reported toothache in the past 6 months [11]. Therefore, it is reasonable to assume that the most prevalent negative impact is physical rather than psychological in nature [2]. In countries with a low occurrence of toothache, loss of teeth, or untreated caries, embarrassment when smiling is one of the most prevalent impacts, suggesting that the epidemiological profile influences the pattern of negative impacts of oral health conditions on quality of life [2, 6].

 The *quilombola* and non-*quilombola* adolescents presented differences in the occurrence of tooth brushing discomfort. A previous study found that the two populations did not differ significantly as regards to other aspects related to oral health conditions, including toothache, oral health self-assessment, and need for dental treatment. However, differences in the use of dental services were found [11]. Therefore, given that *quilombola* adolescents had a lower prevalence of dental consultations at some point in their lives, and that tooth brushing is a technique that must be taught [27], it is acceptable to assume that different oral hygiene techniques can influence tooth brushing discomfort, as well as other oral clinical conditions not yet investigated.

The prevalence of at least one negative impact increased with age in the total and *quilombola* samples. Previous studies found a direct relation between dental cavities and increasing age during adolescence, which is a very risky period for oral and general health [28]. Therefore, poorer oral health conditions increase the probability of negative impact on quality of life among older adolescents [29].

In the present study, the adolescents who mentioned feeling lonely sometimes in the last 12 months presented a higher prevalence of negative impact of oral health conditions on daily life, except among *quilombola* adolescents. The feeling of loneliness was used as an indicator of the mental health status of adolescents. Studies have shown that individuals who had poor social support and who lived alone adopted fewer healthy preventive behaviours and had a higher prevalence of oral diseases, probably because they feel less motivated to perform self-care [30, 31].

The worst self-assessment for oral health and the perception of the need for dental treatment were associated with negative impact of oral health conditions on daily life in the total and *quilombola* samples. This relation was expected, as there is a match between clinical condition and self-perception of oral health, especially in the more painful and esthetical cases. Damage caused by oral problems negatively affect quality of life, self-esteem, and oral health perception [32, 33].

Toothache is an important factor associated with the negative impact of oral health conditions on quality of life [2, 3, 7, 25]. In the present study, both the *quilombola* and non-*quilombola* adolescents who had toothache in the previous 6 months had a higher prevalence of negative impacts. Toothache is considered as an indicator of the prevalence of carious lesions and is closely associated with negative impact of oral health conditions on the quality of life [34]. Various studies have reported an inverted correlation between the number of teeth without cavities and the negative impact on daily activities due to oral problems [2, 21, 35]. As toothache is a common health problem around the world, its monitoring could be used as a strategy for surveillance of the population’s oral health. The prevalence of toothache in adolescents is directly related to the quality of national oral health services, and the reduction of its occurrence is part of the Global Goals for Oral Health 2020 of the World Health Organization [36, 37].

Among the associations that were not observed in the present study, gender and economic status stand out, which were shown in other studies with Brazilian urban adolescents [2, 18, 29]. These studies found a higher prevalence of a negative impact from oral conditions on quality of life among girls, which the authors explained could be related to greater self-criticism regarding dental appearance and low self-esteem among girls and/or stereotypes of masculinity which contributed to greater resistance to pain among boys [2, 18, 29]. Thus, cultural distinctions related to gender may contribute to this association not being observed among rural adolescents. Regarding economic status, the majority of the population was in the lowest level. This homogeneity may have contributed to the lack of association with this variable in the study.

Moreover, differences in the occurrence of at least one negative impact of oral conditions on quality of life were not found between *quilombola* and non-*quilombola* adolescents, despite differences in associated factors. *Quilombola* adolescents face social and health inequities, including more difficult access to dental services [11]. Geographical distance, transportation from *quilombola* communities to health services, the restricted schedule of health services, the prioritisation of services to other population groups, and the need of a legal representative for dental services for adolescents all make it difficult for the oral health of these adolescents to receive due attention [38].

Despite its relevance for the awareness of oral health conditions’ impact on the lives of *quilombola* and non-*quilombola* rural adolescents, the present study has some limitations. These are due to the lack of information on the clinical conditions of adolescents and the impossibility of inferring the temporality of some observed associations because of the cross-sectional nature of the study. Moreover, because the sample was not primarily designed to test differences between *quilombolas* and non*-quilombolas*, there may not have been enough sampling power for some variables.

## Conclusion

The negative impact of oral health conditions on quality of life was reported by a great number of the *quilombola* and non-*quilombola* rural adolescents. The factors associated with the prevalence of occurrence of at least one negative impact of oral conditions on quality of life were different between *quilombola* and non-*quilombola* adolescents, reinforcing the need to treat *quilombola* adolescents as a separate group.

The characteristics related to dental care, such as worst oral health self-assessment, perception of the need for dental treatment, and occurrence of toothache, were associated with occurrence of negative impact of oral health conditions on daily life. This result reinforces the need to improve attention and support for the oral health of *quilombola* and non-*quilombola* rural adolescents through preventive measures, monitoring, and treatment of oral diseases. It is also necessary to incorporate strategies that take into consideration the social context in which adolescents live that may influence their oral healthcare [19].

In addition to improving conditions for *quilombola* and non-*quilombola* adolescents to access dental services, we highlight the need for public policies to improve living conditions in rural communities, such as permanent and adequate access to fluoridated water. Moreover, intersectoral health education activities involving adolescents and their families should be carried out in partnerships among health services, schools, and community organizations. These activities should focus on cultural aspects, popular knowledge, and strengthen the concept of oral health as a basic human right and not as a privilege.

Oral health policies in Brazil continue to focus more heavily on oral rehabilitation and tooth extraction than on preventive approaches to oral health. Oral health transcends odontology. Therefore, oral health initiatives need to be comprehensive e and intersectoral to allow for holistic attention to health that considers the needs of different population groups [32].

## Data Availability

The datasets used and analysed in the current study are available upon request to the corresponding author.

## Abbreviations

PR: Prevalence ratio
OIDP: Oral impact on daily performance
BMI: Body mass index
